# *Interleukin-6* gene −572 G > C polymorphism and myocardial infarction risk

**DOI:** 10.1515/med-2020-0407

**Published:** 2020-05-15

**Authors:** He-guo Ding, Yan-wei Yin, Sun-lin Liu

**Affiliations:** Department of Respiration, Huzhou 3rd Hospital, 2088 Tiaoxi Road, Huzhou, Zhejiang 313000, China; Department of Emergency, Chinese PLA Air Force General Hospital, Beijing, China

**Keywords:** interleukin-6, myocardial infarction, polymorphism, meta-analysis

## Abstract

**Introduction:**

The association between *interleukin-6* (*IL-6*) gene −572 G^C polymorphism and myocardial infarction (MI) risk has not been established. We adopted this meta-analysis for further insight into the case–control studies.

**Materials and methods:**

To investigate the genetic association, we searched multiple databases, including Web of Science, EMbase, CBM disc, PubMed and CNKI. Also, we manually identified the searched references. All the statistical analyses were conducted using Stata 11.0.

**Results:**

A total of five studies were identified, involving 2,526 MI cases and 3,027 controls. The results revealed a significant association between *IL-6* gene −572 G^C polymorphism and MI, implying that the *IL-6* gene −572 C allele may be a protective factor for MI (for C allele vs K allele: OR = 0.85, 95% CI = 0.73–0.99, *p* = 0.041; for C/C vs G/G: OR = 0.55, 95% CI = 0.31–0.98, *p* = 0.044; for C/C vs G/C + G/G: OR = 0.60, 95% CI = 0.41–0.89, *p* = 0.011). However, in the subgroup analysis with regard to ethnicity, no significant correlation was identified between *IL-6* gene −572 G^C polymorphism and MI among Europeans.

**Conclusion:**

The *IL-6* gene −572 C allele may be a protective factor for MI. Future studies involving larger sample bases are still recommended.

## 1 Introduction

Myocardial infarction (MI) is one of the most fatal factors in the realm of noncommunicable diseases around the world. MI is caused mainly by the interruption of the blood supply in the heart, which may cause myocardial necrosis. Although a combined impact from environmental and genetic factors may play a critical role [1,2], the explicit mechanism underlying MI remains unclear. It is well known that inflammation is an essential part of the pathological process in atherosclerosis and its clinical result MI [3,4,5], suggesting that inflammatory cytokines act as risk factors for MI.

Interleukin-6 (IL-6), a proinflammatory cytokine, has a molecular weight of around 26 kD [6], and it plays an important role in host defense through activated macrophages and lymphocytes. The encoded *IL-6* gene is located on the chromosome 7p21, spanning about 5 kb, consisting of five exons and four introns [6,7]. According to previous researches, polymorphisms in the IL-6 promoter region, in particular −572 G^C, are associated with transcription of *IL-6* gene and its secretion [8,9]. Moreover, IL-6 transcripts are present in the atherosclerotic arterial wall [10,11], and serum IL-6 is elevated in patients with MI [12]. Ridker et al. demonstrated that serum IL-6 was associated with an increased risk of MI in an apparently healthy population [13]. Thus, *IL-6* gene −572 G^C polymorphism may play a significant role in the development of MI.

Within the past few years, the role of *IL-6* gene −572 G^C polymorphism in human atherosclerotic disease leading to MI progression has been widely studied; however, the result is uncertain. To the best of our knowledge, few studies have managed to reveal a significant association between *IL-6* gene −572 G^C polymorphism and MI risk [14,15,16,18]. However, Wei et al. revealed a close correlation between the *IL-6* gene −572 G allele and the elevated risk of MI [17]. Therefore, to have a better understanding on this issue, we performed this meta-analysis using all available case–control studies to evaluate the association between the *IL-6* gene −572 G^C polymorphism and the risk of MI.

## 2 Materials and methods

### 2.1 Data sources

This meta-analysis is consistent with the systematic review and meta-analysis (PRISMA) standards for priority reporting projects [19]. A comprehensive computer-based search of Web of Science, Embase, PubMed, CD-ROM Chinese biomedical literature analysis and retrieval system (CBMdisc) and Chinese knowledge network (CNKI) (as of June 15th, 2015) was performed to filter the related studies. The following keywords were used for searching: (“interleukin-6” OR “IL-6”) AND (“polymorphism” OR “mutation” OR “variant” OR “genotype”) AND (“myocardial infarction” OR “MI” OR “coronary heart disease” OR “CHD” OR “coronary artery disease” OR “CAD”). These literature searches were only performed on articles in English and Chinese. In addition, to meet the inclusion criteria for the literature, references were retrieved to identify additional references if ED was uncertain initially.

### 2.2 Inclusion criteria

The study of the combined meta-analysis must conform to the following criteria: (1) human studies, (2) studies on the relationship between *IL-6* gene −572 G^C polymorphism and MI, (3) independent case–control studies, (4) adequate published data on the genotypes or allele frequencies for estimating odds ratio (OR) with 95% confidence interval (CI) and (5) unpublished data.

### 2.3 Data extraction

Data were independently analyzed by two authors (Ding HG and Yin YW), and the results were reviewed by the third author (Liu SL). Multiple items of information were derived and analyzed from each publication: first, the author’s surname, publication year, country and race, locus of control, case number and control number, genotype and allele information. In addition, Hardy Weinberg was also collected (*p* < 0.05 of HWE was considered as statistically significant).

### 2.4 Quality score assessment

Ding HG and Yin YW independently assessed the quality of the included studies using the Newcastle–Ottawa Scale (NOS), which is applicable for both case–control and cohort studies [20]. We assessed the study from three aspects (selection, comparability and exposure), and each satisfactory answer was scored a star. The NOS range is from zero (worst) to nine stars (best). Studies with a score greater than 7 were considered as high quality. Liu SL examined the results, and further discussion was required when there was disagreement among investigators.

### 2.5 Statistical analysis

The strength of the association between *IL-6* gene −572 G > C polymorphism and MI risk was measured by ORs with 95% CIs. The pooled ORs were estimated for four genetic models (allelic model: C allele vs G allele, additive model: C/C vs G/G, recessive model: C/C vs G/C + G/G, and dominant model: C/C + G/C vs G/G). Heterogeneity between studies was formally tested by using Cochran’s *Q* statistic and considered statistically significant when *p* < 0.10. Heterogeneity was also measured with *I* ^2^ statistic (*I* ^2^ > 50% indicated evidence of heterogeneity) [21,22]. The fixed-effects model was used in the absence of between-study heterogeneity; otherwise the random-effects model was used [23,24]. The subgroup analysis was performed based on ethnicity. Furthermore, to evaluate the stability of the results, the sensitivity analysis was performed by limiting the meta-analysis to studies conforming to HWE. An estimate of potential publication bias was assessed with Begg’s funnel plot and Egger’s regression test (*p* < 0.05 was considered representative of statistically significant publication bias) [25]. Data from the meta-analysis were analyzed using Stata 11.0 (StataCorp LP, College Station, TX).

## 3 Result

### 3.1 Study characteristics

**Figure 1:**
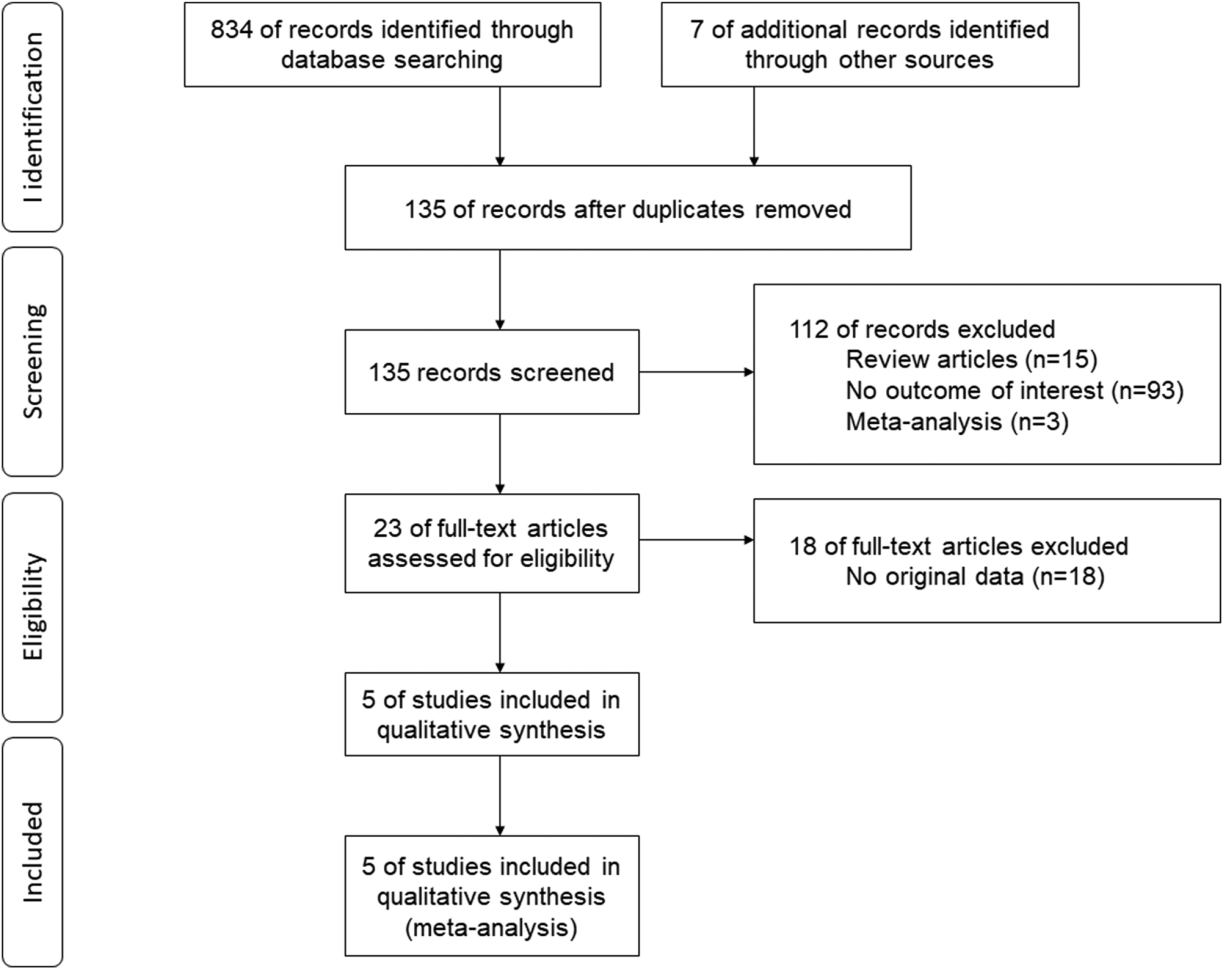
Flow diagram of the study selection process.

The study logistics are shown in Figure 1. A total of five eligible studies were selected [14,15,16,17,18], which include 2,526 MI cases and 3,027 controls. Table 1 presents an overview of the main characteristics of the study. Four studies were conducted on Europeans [14,15,16,18], and one study was performed on Asians [17]. The study subjects were from China, France, Sweden, Turkey and United Kingdom. The genotype distributions among the controls of all studies followed the HWE except the study by Coker et al. [18] (*p* < 0.05). The average score of the NOS results was 8 and was relatively acceptable.

**Table 1:** Characteristics of studies included in this meta-analysis

First author	Year	Country	Ethnicity	Source of control	Sample size (case/control)	Genotypes distribution (case/control)					HWE Y/N(P)	Score
						G/G	G/C	C/C	G	C		
Georges [14]	2000	France	European	PB	611/665	552/589	58/73	1/3	1,162/1,251	60/79	Y (0.650)	7
Bennet [15]	2003	Sweden	European	PB	1,115/1,435	1,038/1,327	74/104	3/4	2,150/2,758	80/112	Y (0.201)	8
Kelberman [16]	2004	UK	European	PB	505/547	433/475	71/69	1/3	937/1,019	73/75	Y (0.774)	8
Wei [17]	2005	China	Asian	PB	128/145	6/1	50/43	72/101	62/45	194/245	Y (0.115)	8
Coker [18]	2011	Turkey	European	PB	167/235	126/169	30/45	11/21	282/383	52/87	N (0.000)	9

PB: population-based.

HWE: Hardy–Weinberg equilibrium, Y: yes, N: no.

### 3.2 Quantitative synthesis

**Figure 2:**
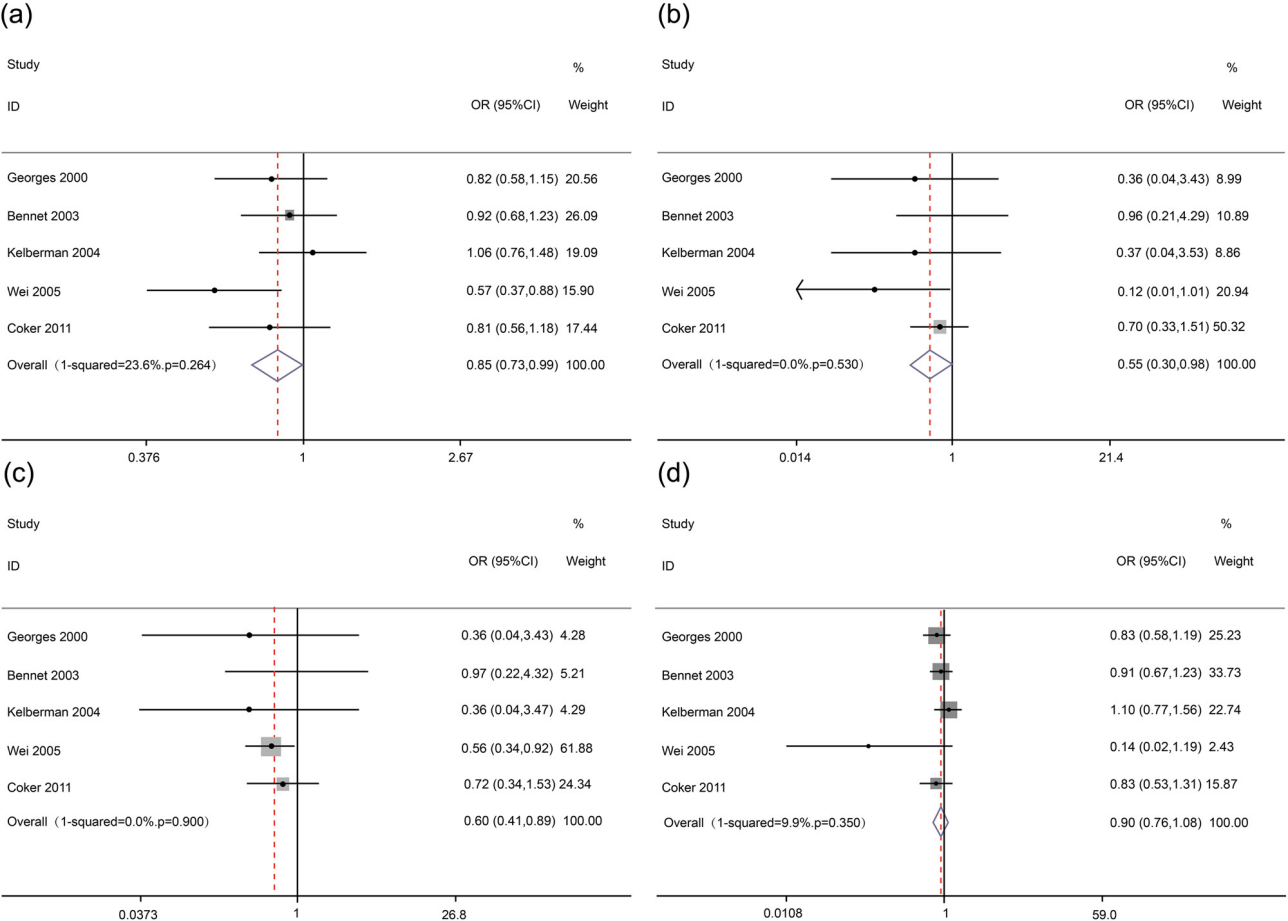
Forest plots for *IL-6* gene −572 G > C polymorphism and MI in overall analysis. (a) (allelic model: C allele vs G allele), (b) (additive model: C/C vs G/G), (c) (recessive model: C/C vs G/C + G/G) and (d) (dominant model: C/C + G/C vs G/G).

Given that no heterogeneity existed in overall comparisons (for C allele vs G allele: PQ = 0.264, *I* ^2^ = 23.6%; for C/C vs G/G: PQ = 0.530, *I* ^2^ = 0%; for C/C vs C/G + G/G: PQ = 0.900, *I* ^2^ = 0%; for C/C + C/G vs G/G: PQ = 0.350, *I* ^2^ = 9.9%), we used the fixed-effects model to calculate the pooled ORs. The overall results showed that there was a significant association between *IL-6* gene −572 G > C polymorphism and MI, suggesting that *IL-6* gene −572 C allele may be a protective factor for MI (for C allele vs G allele: OR = 0.85, 95% CI = 0.73–0.99, *p* = 0.041; for C/C vs G/G: OR = 0.55, 95% CI = 0.31–0.98, *p* = 0.044; for C/C vs G/C + G/G: OR = 0.60, 95% CI = 0.41–0.89, *p* = 0.011; for C/C + G/C vs G/G: OR = 0.90, 95% CI = 0.76–1.08, *p* = 0.250). The main results of the meta-analysis are presented in Table 2 and Figure 2.

**Table 2:** Meta-analyses of IL-6 gene −572 G > C polymorphism and MI in each subgroup

Category	Sample size (case/control)	C vs G		C/C vs GG		C/C vs G/C + G/G		C/C + G/C vs G/G	
		OR (95% CI)	*P* a	OR (95% CI)	*P* a	OR (95% CI)	*P* a	OR (95%CI)	*P* a
Overall	2,526/3,027	0.85 [0.73, 0.99]	0.264	0.55 [0.31, 0.98]	0.530	0.60 [0.41, 0.89]	0.900	0.90 [0.76, 1.08]	0.350
Europeans	2,398/2,882	0.90 [0.76, 1.07]	0.680	0.66 [0.35, 1.23]	0.850	0.67 [0.36, 1.25]	0.840	0.92 [0.77, 1.10]	0.690

Sensitivity analysis									
BH	2,359/2,792	0.86 [0.72, 1.02]	0.160	0.39 [0.15, 0.99]	0.460	0.57 [0.36, 0.89]	0.850	0.91 [0.75, 1.11]	0.230

BH: based on HWE (studies without HWE were excluded).

^a^ *P* values for heterogeneity from *Q*-test.

However, in the subgroup analysis by ethnicity, no significant association was found between *IL-6* gene −572 G > C polymorphism and MI among Europeans (for C allele vs G allele: OR = 0.90, 95% CI = 0.76–1.07, *p* = 0.230; for C/C vs G/G: OR = 0.66, 95% CI = 0.35–1.23, *p* = 0.190; for C/C vs G/C + G/G: OR = 0.67, 95% CI = 0.36–1.25, *p* = 0.210; for C/C + G/C vs G/G: OR = 0.92, 95% CI = 0.77–1.10, *p* = 0.360). We did not perform the subgroup analysis for Asians as only one study involved Asians. The main results of the meta-analysis are summarized in Table 2.

### 3.3 Sensitivity analysis

The sensitive analysis was used to evaluate the stability of the results. In the study by Coker et al., the sensitivity analysis was excluded due to the deviation of the genotype distribution in the control group from HWE. We found that the corresponding pooled ORs were not significantly altered in the recessive model and the dominant model. In contrast, the corresponding pooled ORs were significantly altered in the allelic model and the additive model, indicating that the study without HWE should be viewed as an influencing factor on the overall results. Table 2 presents the results of sensitivity analyses.

### 3.4 Publication bias

**Figure 3:**
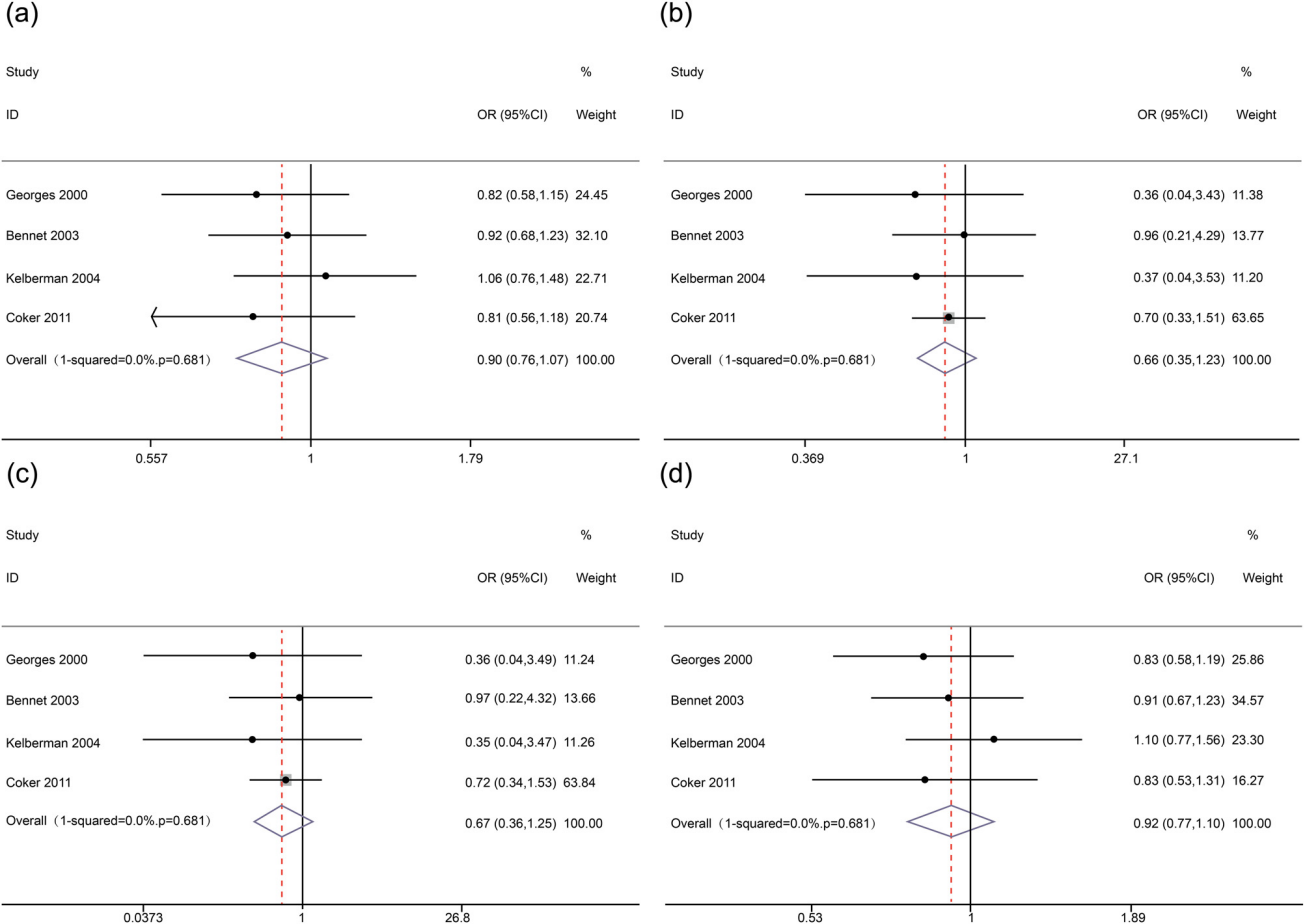
Forest plots for *IL-6* gene −572 G > C polymorphism and MI in the subgroup analysis. (a) (allelic model: C allele vs G allele), (b) (additive model: C/C vs G/G), (c) (recessive model: C/C vs G/C + G/G) and (d) (dominant model: C/C + G/C vs G/G).

Begg’s funnel plot and Egger’s regression test were performed to assess the publication bias of the studies. No obvious asymmetry was observed in any genetic model according to the visual assessment of the funnel plot (Figure 3). Furthermore, Egger’s regression test also suggested that there was no publication bias in the present meta-analysis (*p* = 0.118 for allelic model, *p* = 0.221 for additive model, *p* = 0.860 for recessive model and *p* = 0.083 for dominant model).

## 4 Discussion

MI is a complex disease associated with multiple factors including environmental and genetic impacts [1,2]. The association between *IL-6* gene −572 G > C polymorphism and MI has been extensively studied. However, previous studies exploring the existing evidence were most frequently performed as case–control studies [14,15,16,17,18], lacking statistical power to reveal the association between the *IL-6* gene −572 G^C polymorphism and MI. Recently, the meta-analysis has been widely utilized in genetic studies because it is capable of detecting small effects between gene polymorphism and human disease [26,27,28,29]. By enlarging the study sample, the meta-analysis is statistically convincing enough to explore the underlying correlation of the gene polymorphism with human disease. The previous meta-analysis including 6,076 MI cases and 5,645 controls showed that *IL-6* gene −174 G^C polymorphism is not associated with the elevated risk of MI [30]. Here, our key focus is on the association between *IL-6* gene −572 G^C polymorphism and MI, and this meta-analysis is used to provide a more precise validation.

As far as we know, this is the first comprehensive meta-analysis to date whose main purpose is to explore the relationship between *IL-6* gene −572 G^C polymorphism and MI. In this meta-analysis, the integrated evidence indicates that the *IL-6* gene −572 C allele may play a role as a protective factor for MI. Given that potential ethnic differences might have an indirect impact, we also performed the subgroup analysis with regard to ethnicity. The results show that among Europeans there is no significant association between *IL-6* gene −572 G^C polymorphism and MI. The inconsistent results for Europeans in the subgroup analysis may partially result from the genetic diversity among ethnicities. MI is a complex disease; in addition to genetic factors, environmental factors also have certain impacts in MI etiology. Thus, this discrepancy may also be caused by varied geographic distribution, linked to climate, diet, financial status and living conditions. For Asians, we did not perform the subgroup analysis because of insufficient studies. Furthermore, considering that the genetic correlation results from the case–control study may be biased, the genotype distribution in the control group deviated from HWE [31]. We also performed the sensitivity analysis restricted to the studies conforming to HWE. It is shown that the corresponding pooled ORs were significantly altered in the allelic model and the additive model, implying that conforming to HWE should be considered as an influencing factor on the overall results. However, the results of the sensitivity analysis further strengthened our conclusion that the *IL-6* gene −572 C allele may be a protective factor for MI.

**Figure 4:**
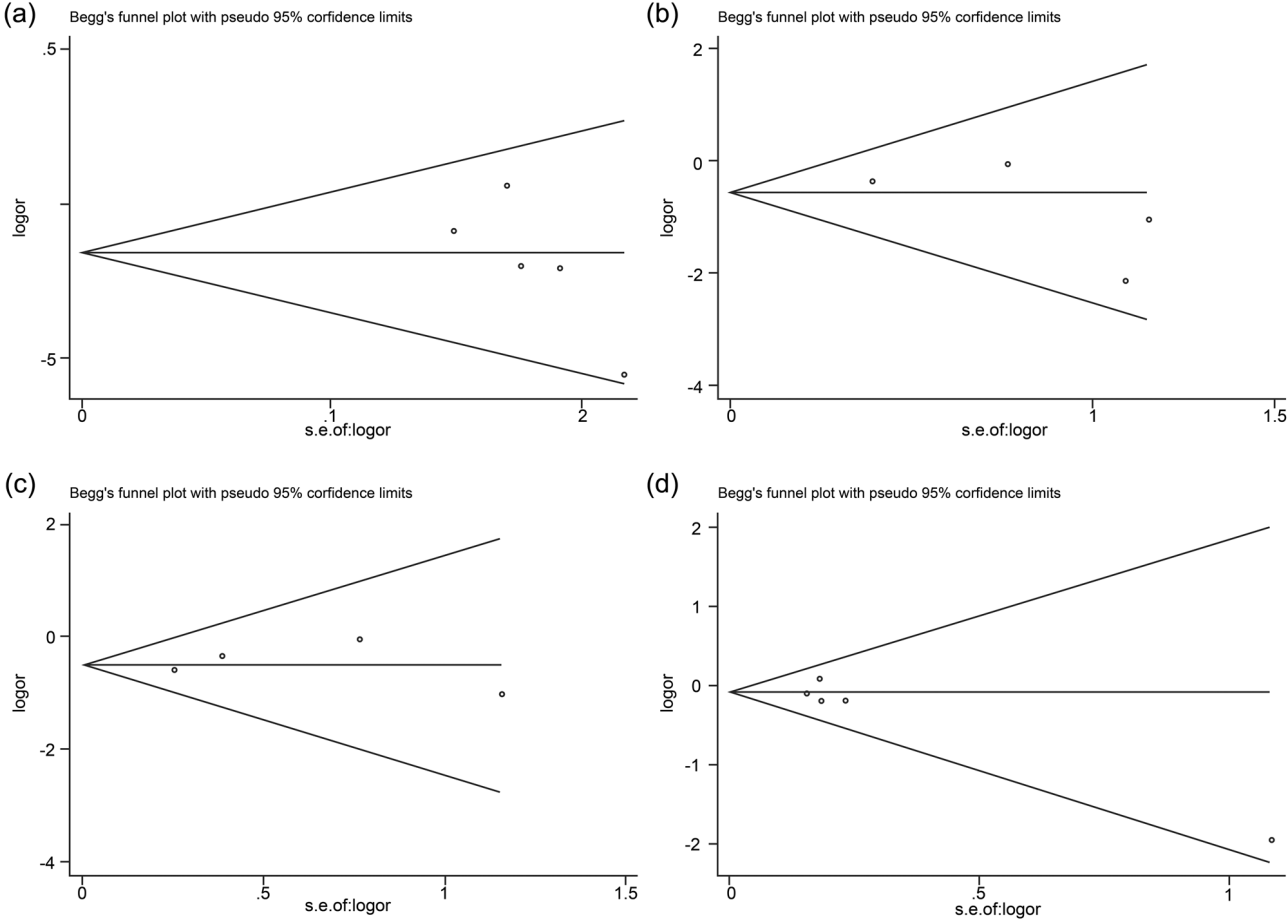
Funnel plots for *IL-6* gene −572 G > C polymorphism and MI in overall analysis. (a) (allelic model: C allele vs G allele), (b) (additive model: C/C vs G/G), (c) (recessive model: C/C vs G/C + G/G) and (d) (dominant model: C/C + G/C vs G/G).

There is no heterogeneity and publication bias in the overall comparisons; in other words, the present meta-analysis was statistically validated and reliable. However, the potential limitations of this study should not be ignored. First, this meta-analysis focused merely on studies reported in English and Chinese. Thus, publications written in other languages may not be fully consistent with the present results; however, in our present work, no obvious biases were observed in the funnel plots or Egger’s regression test. Second, the number of studies and the number of subjects included in this meta-analysis are relatively small, and only Europeans were analyzed in the subgroup analysis as only one study was performed in Asians, which could increase the probability of false positives. Taking into consideration that potential ethnic differences might exist in the distribution of genotypes and the allele frequencies, it is a weakness of this study due to the failure to analyze the association between *IL-6* gene −572 G^C polymorphism and MI among Asians. Therefore, the results should be interpreted with caution. Third, meta-analysis has its own inherent limitations; its retrospective nature is associated with the deficiencies of the methodology (Figure 4).

In conclusion, our meta-analysis including 5,553 subjects suggests that the *IL-6* gene −572 C allele may be a protective factor for MI. However, we should view the results in an objective manner as there are still some limitations. In addition, the sample size (the number of studies and subjects) in this meta-analysis is relatively small. Further studies with enlarged enrollment of study subjects, e.g., with multicenter case–control studies, will be required to verify our findings within the near future.
